# Gender and active travel: a qualitative data synthesis informed by machine learning

**DOI:** 10.1186/s12966-019-0904-4

**Published:** 2019-12-21

**Authors:** Emily Haynes, Judith Green, Ruth Garside, Michael P. Kelly, Cornelia Guell

**Affiliations:** 1 European Centre for Environment & Human Health, University of Exeter Medical School, Truro, UK; 2 Primary Care and Public Health Sciences, King’s College London, London, UK; 3 Cambridge Institute of Public Health, University of Cambridge, Cambridge, UK

**Keywords:** Active travel, Gender, Machine learning, Text analytics, Qualitative synthesis

## Abstract

**Background:**

Innovative approaches are required to move beyond individual approaches to behaviour change and develop more appropriate insights for the complex challenge of increasing population levels of activity. Recent research has drawn on social practice theory to describe the recursive and relational character of active living but to date most evidence is limited to small-scale qualitative research studies. To ‘upscale’ insights from individual contexts, we pooled data from five qualitative studies and used machine learning software to explore gendered patterns in the context of active travel.

**Methods:**

We drew on 280 transcripts from five research projects conducted in the UK, including studies of a range of populations, travel modes and settings, to conduct unsupervised ‘topic modelling analysis’. Text analytics software, Leximancer, was used in the first phase of the analysis to produce inter-topic distance maps to illustrate inter-related ‘concepts’. The outputs from this first phase guided a second researcher-led interpretive analysis of text excerpts to infer meaning from the computer-generated outputs.

**Results:**

Guided by social practice theory, we identified ‘interrelated’ and ‘relating’ practices across the pooled datasets. For this study we particularly focused on respondents’ commutes, travelling to and from work, and on differentiated experiences by gender. Women largely described their commute as multifunctional journeys that included the school run or shopping, whereas men described relatively linear journeys from A to B but highlighted ‘relating’ practices resulting from or due to their choice of commute mode or journey such as showering or relaxing. Secondly, we identify a difference in discourses about practices across the included datasets. Women spoke more about ‘subjective’, internal feelings of safety (‘I feel unsafe’), whereas men spoke more about external conditions (‘it is a dangerous road’).

**Conclusion:**

This rare application of machine learning to qualitative social science research has helped to identify potentially important differences in *co-occurrence of practices* and *discourses about practice* between men’s and women’s accounts of travel across diverse contexts. These findings can inform future research and policy decisions for promoting travel-related social practices associated with increased physical activity that are appropriate across genders.

## Background

There is an urgent need for innovative approaches to increase population levels of physical activity, for which interventions have shown only modest success to date [1]. In part, this is because current approaches that aim to enhance physical activity deal poorly with complexity, failing to address the interlinkages and interactions, rather than the mere plurality, of factors and processes. Even multi-level, sophisticated intervention frameworks remain under-theorised and focused on individual behaviour [2].

Globally, there is an increasing acknowledgement that multi-sectoral approaches are needed to encourage people to walk and cycle as incidental forms of physical activity that can be integrated into the day [3–7]. However, much is still to be learned about the conditions for change of transport, travel or mobility [8], and how these might change across population groups. Our particular interest here is in gender. There is a growing evidence base for gender differences in the way that men and women travel, the length and composition of their commute [4, 9, 10], the purpose of daily travel [11, 12], preferred travel modes and the contextual factors that construct these preferences [13, 14]. Even the impact of key life events (such as the birth of a baby) on travel behaviour differs between men and women [15, 16]. However, more nuanced research trials and evidence syntheses are called for to explore the independent effect of gender on travel behaviour and the significance of such on policy development [14].

To develop more appropriate insights for the complex challenge of increasing population levels of activity, recent research has drawn on social practice theory. Social practices are complex sets of activities, shaped by social, political and economic contexts, and transmitted across time and space [17]. A small but growing body of in-depth qualitative studies on ‘social practice’ contribute novel perspectives to the public health field [18–20], including studies of physical activity, which have explored the recursive and relational character of active living and interrelated social practices [21]. However, qualitative accounts are inherently context specific, small in scale and, for the interest of this paper, have for the most part not specifically explored gender differences. Approaches to synthesising qualitative evidence have mostly focused on integrating the findings from published research, for example in meta-ethnographies [22]. However, qualitative evidence synthesis requires careful considerations of the context in which data have been collected, analysed and framed. Similarly, secondary analysis and synthesis of primary qualitative data risks losing the connection to the original data collection, analysis and context. With a call in the public health community for ‘less research and more thinking’ [23], there is a need for efficient and thoughtful methods for synthesising primary qualitative data; the methods explored in this case-study are based on the premise that some approaches to quantitative evidence synthesis may be useful for synthesising large qualitative datasets.

For our focus of interest, active travel as a social practice, we have started to cumulate rich qualitative evidence on travel, transport or mobility practices in a range of contexts and population groups across the United Kingdom. These datasets include gender descriptors. In the original studies, most respondents had reported their gender as either ‘male’ or ‘female’, and the analysis is of those data. We ask what general learnings about the conditions for change come from synthesising and comparing data from these context-specific studies, without losing sight of the context in which these data were collected and made sense of. Our aim was to explore gendered patterns between practice connections in the context of active travel by pooling and synthesising insights from individual studies about travel, without losing sight of the explanatory strength of qualitative data and its social theoretical framing or original context.

## Method

### Research design

We worked with primary data, in the form of anonymised textual transcripts, from five research projects (that included seven separate qualitative datasets) (Table 1). The investigations ranged from commuting in Cambridge (three qualitative datasets conceived, collected and analysed by different researchers) [21, 24, 25], cycling in London [26], free bus passes for young people in London [27], to the impact of a new motorway in Glasgow [28] and an evaluation for a proposed Graduated Driver’s Licence Scheme in Northern Ireland [29]. We chose these research projects because some of the authors of this paper were directly involved in the conception, data collection and/or analysis of the original data, and were therefore familiar with the context of each individual dataset and had approached the research from a similar epistemological stance as we intended in this synthesis. This familiarity was important given the novelty of our analytical method.

**Table 1:** Demographic information

Study	Study code	Participants (n)	Gender (F;M)	Age (years)	Transcripts (n)		
					*Total*	*Interview(I)*	*Focus group (FG)*
Commuting in Cambridge	CO	115	70; 45	18–70	115	115	0
	*COA*	*49*	*30;19*	*20–69*			
	*COB*	*28*	*19;9*	*20–59*			
	*COC*	*38*	*21;17*	*30–79*			
Traffic in Glasgow	MG	30	19;11	20–35	30	30	0
Cycling and the City	CC	78	67;11	18–64	70	69	1
On the Buses^a^	OB	166	96;70	12–18 60+	44	33	11
Graduated Driver’s Licence^b^	YD	84	36;34	16–21 (plus parents	21	0	21
Total					**280**		

^a^ On the Buses: Evaluating the impact of free bus travel for young people on the public health

^b^ Pre-intervention qualitative component of proposed evaluation of public health impacts of Graduated Driver Licencing in Northern Ireland

All five research projects were conducted in the UK, across a range of settings (including the cities of Belfast, London, Glasgow, Cambridge and Cardiff, and rural areas) and populations. Participants in the pooled dataset included men and women aged 12 to over 80 years. The primary data was collected between 2010 and 2016. Each primary study collected data via interview and/or focus groups that were audio recorded and transcribed verbatim. We edited a total of 280 transcripts into a standardised format for the analysis. Anonymised transcripts were password protected and shared and stored only within secured platforms. Ethical approval was granted by the original bodies where re-use had not been pre-approved and was overseen by the University of Exeter Ethics Committee.

### Analysis

#### Stage one

We analysed the pooled dataset in two stages. We undertook the first stage of the analysis using an unsupervised machine learning approach. This was applied via the text analytics software tool, Leximancer (Version 4.51). The detailed rationale for applying Leximancer in this study and a step-by-step guide for doing so are described elsewhere [30]. In short, Leximancer applies a form of text mining that mostly utilises a statistical unsupervised machine learning approach. The software performs an automatic unsupervised analysis of text documents which are imported as individual files or folders, to identify not only lists of key concepts, but concepts in context [31]. The approach is entirely based within the text and requires little input from the researcher and no a priori rules or training sets. This is different to researcher-driven approaches and other pattern-recognition software such as NVivo. Complex algorithms are drawn upon to identify word- and name-like terms and, innovatively, determine interconnections, structures and patterns between terms to develop ‘concepts’ in context. The software is able to quantify the inter-relationships amongst concepts, including how frequently different concepts occur, how they inter-relate with each other, and also in what contexts they inter-relate. This unsupervised analysis of inter-relating terms or ‘concepts’ is known as ‘topic modelling analysis’ and holds the potential for identifying new and connected concepts within pooled datasets, as well as expediting the early stages of qualitative analysis process.

It is important to note that there is a disconnect between the language used within the fields of text analytics and traditional qualitative analysis [30]. These differences are defined in Table 2. For the purpose of this case study, we use Leximancer terminology to report the findings.

**Table 2:** Glossary of terms used by Leximancer vs. qualitative analysis

Leximancer terminology	Description	Common qualitative thematic analysis terminology	Description
Term	Words in the text that have been examined for frequency of co-occurrence with other words and synonyms from the thesaurus and are weighted or scored according to evidence that a concept is present in a sentence.	Content code	Basic unit of meaning.
Concept	Collections of words or ‘terms’ that travel together within the text. They are parent terms that have been identified through semantic and relational word extraction that share similar meaning and/or space within the text.	Sub-category	Descriptive family of codes
Theme	Emergent concept groups that are highly connected, parent concepts.	Category	Defining or conceptual labels for family of codes.
		Theme	Overarching category based on interpretive, theoretical, conceptual insight of researcher.
‘Important’	The hierarchy of ‘importance’ indicates concept connectedness.	Meaningful, interpretive, important	Basis for theoretical understanding of the data; particularly pertinent or revealing in relation to the research question.

We imported the 280 transcripts into Leximancer for analysis. The only information that we gave the software was the gender of the respondent and the original study of each transcript. The six steps involved in this first stage of the analysis were conducted by one member of the research team (EH) and can be found in the Additional file 1: Table A, and discussed in greater detail in a separate publication [30].

We present the findings of the analysis in two ways. A conceptual map (or inter-topic distance map), which provides a “bird’s eye view” of the semantic data, and a quantitative data summary of the data as frequency counts. On the map, the major themes are illustrated as coloured bubbles and within the bubbles are collections of inter-linked dots which are the constituent concepts that make up that theme. The proximity of the bubbles or concept dots to one another indicate conceptual similarity, with those clustered together most closely related. The bars of the frequency graph are coloured to correspond with the bubbles of the conceptual map to provide an integrative summary of the quantitative and semantic data. Each theme links to a list of downloadable excerpts that are generated to provide contextual detail to evidence each automatically identified theme or concept.

The aim of applying a machine learning approach to our data was to *uncover networks or patterns* that had not emerged from the original and more traditional forms of qualitative data analysis of the individual datasets. However, despite the utility of Leximancer in these early stages of concept identification and data coding, researcher-led interpretative work remained an essential component of the analysis and researcher insight fundamental to developing meaningful outputs [30].

#### Stage two

To make sense of the automatically generated outputs, we applied a second interpretive stage to the analytical process, which required researcher-led, in-depth interpretation of the data in the light of our aim. In this stage, we were guided by social practice theory to explore explicit and implicit practices behind the automatically generated themes, concepts and connections. We conducted a series of ‘queries’ in Leximancer in response to potential lines of enquiry stimulated by stage one and our theoretical interest. These queries produced a compilation of all text examples from which the themes and concepts of interest had been generated. One researcher (EH) coded the excerpt lists, discussed theoretical assumptions with other members of the research team and reported the findings as interpretive themes.

## Findings

**Fig. 1:**
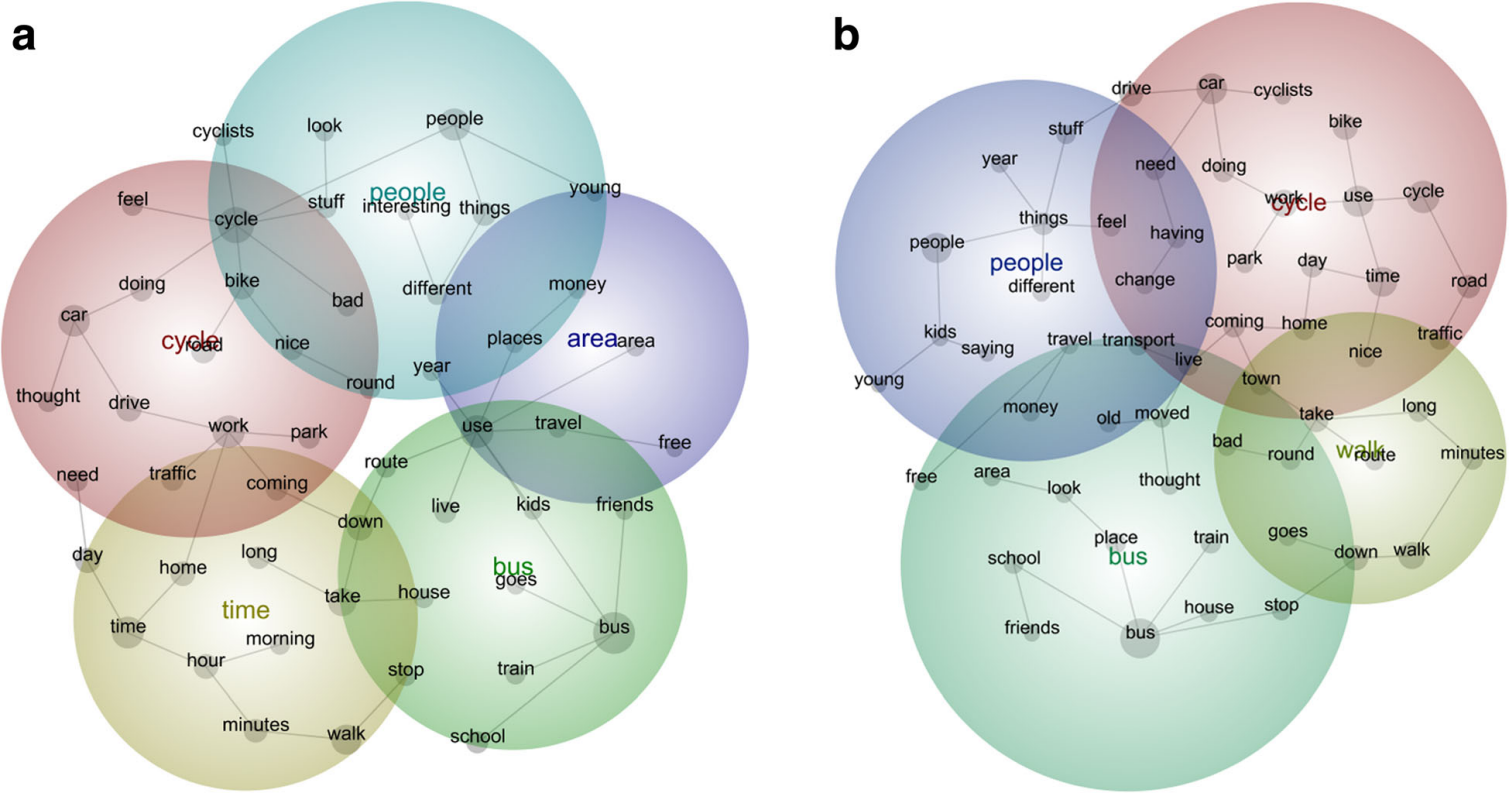
Inter-topic distance maps from data analysis of women (1**a**) and men (1**b**)

Semi-automated text analytics allowed us to explore gendered accounts of travel. Here we present the software outputs as inter-topic distance maps for women (Fig. 1a) and men (Fig. 1b) and the list of automatically identified concepts (Additional file 2: Table B) in stage one, and our interpretive exploratory analysis in Stage Two.

### Stage one: software outputs; quantifying relational concepts

Figure 1a and b illustrate the concepts and themes automatically identified within the women’s and men’s datasets (shown capitalised in text). Concepts, or co-occurring terms are expressed as grey dots which vary in size, to represent frequency of co-occurrence (the sum of co-occurrence with all other concepts identified), and closeness to other concepts, to represent context. The coloured bubbles are considered ‘themes’, derived from word stems and are named after the most connected concept within the bubble. They are heat mapped to indicate importance (whereby red depicts the most important/interconnected theme, then orange and so on according to the colour wheel) and the resolution or ‘granularity’ can be changed (whereby several narrow themes (high granularity) can be collapsed into fewer broad themes (low granularity). For this analysis, the theme granularity was set at 66% to provide a manageable number of predominant themes for the second phase of the analysis. For women, five themes were displayed; Cycle, Time, Bus, People and Area (Fig. 1a). For men, four themes were Cycle, Walk, Bus and People (Fig. 1b).

The automated analyses identify 51 and 55 concepts within the women’s and men’s accounts. Table B (Additional file 2) presents the first 25 of these concepts. These ‘concepts’ are word stems mentioned frequently in the transcripts (count) together with other frequently used words (relevance). The ten most ‘frequent’ and ‘relevant’ used concepts include all the travel modes, Bus, Cycle, Car, Walk and Drive. Other concepts include, places to travel to or from such as Work, School and Home, logistics such as time, hours, minutes, things, as well as those they travel with or others who travel (people). Most strikingly, the list does not display marked differences between women and men: 96 (91%) of automatically identified concepts occurred in both gender subgroups (Additional file 2: Table B).

### Stage two: researcher-led interpretation; making sense of the maps

We applied a pragmatic, theory-driven approach to the interpretive phase to make meaning of the outputs. As the software’s ‘themes’ and ‘concepts’ displayed on the maps are not actual conceptual, interpretive or explanatory labels but simply derived ‘in-vivo’ from word stems, we needed to identify what understanding could be taken from co-occurrences of words in the most narrow sense or narrated experiences in the most interpretive sense. To do this, we were guided by key characteristics of social practices, as developed by theorist Pierre Bourdieu, namely that practices are complex sets of activities that are shaped by, and enacted with, other practices, and other people and with socio-cultural meaning [17]. We returned to the original data (the identified text that connected the terms on the maps), and coded the excerpt lists of interest, to determine whether these linguistic terms (which were similar between the men’s and women’s data), held the same meaning or were connected in the same way in the two datasets. We explored what ‘themes’ or ‘concepts’ might connote practices and explored any gendered patterns in the practices themselves (3.2.1) and the discourses about practices (3.2.2).

#### Practices occur with other practices: the commute

In both subgroups the most frequently co-occurring concept with Work was Home (25% likelihood of co-occurrence for women and 23% for men), and we decided to treat this connection as describing the social practice of commuting – travelling from home to work and back. In the text extracts that connect Home and Work, women talked about doing other things on route from home to work or vice versa. Talk about these ‘travel diversions’ was evident across all seven datasets and included practices such as shopping, the school run or meeting friends.

‘*I tend to just do all my shopping on the way home from work*.’(*MGIF12; Traffic in Glasgow, Interview, Female, no. 12*).

‘*I usually leave work at about ten to six, I’m usually at the nursery for five to six and then … I usually get home about twenty five to seven, sometimes later’(COAIF18).*

In some cases these necessary diversions were explicitly considered in decisions around travel,

*‘I can see why people don’t use the bus in that respect you know, childcare is a real issue or your childminder isn’t on a bus route or you know.’(COAIF30).*

*‘ … at the end of the day there’s very often something to do that means either going into town or getting to another location which you just simply can’t do on a bike, or picking something up or I don’t know, going to babysit my grandson or something like that and I just have to bring the car then … ’(COCIF21).*

Women also spoke about using the time and space for work; an association not explicitly evident in the men’s data.

‘*I really didn’t, there was no chitchat or anything, I just got my books and highlighter and worked. I never did anything else,*. *.*. *I studied on the bus and it paid dividends because it meant that that was ten hours a week that I wasn’t taking out from being at home with my daughter if you see what I mean*’(COAIF30).

*‘I’m working on something, I’m writing something now, so I might, if I want to quickly look up something, I will do that on the bus. I’ll take the book and find it, or I’ll read a novel which is relaxing.’*(OBIF4).

The commute appears to have many functions for these women. These multi-functional journeys support practice theory in how practices co-occur, are reliant on or shaped by one another and have the potential to act as barriers or facilitators to one another; however in the case of these data, this seems to be evident for women but not for men. Men’s accounts were about linear, rather than multi-functional, journeys, but other associated factors or practices were identified in the men’s talk that could be considered as resulting practices.

*‘And I can probably cycle in my work clothes and might not get too sweaty that I’ve got to start taking a shower and stuff, so once I start to work that through my mind, I can probably just get on and do it really’ (COAIM2; Commuting in Cambridge, Interview, Male, no.2).*

In the same way as the women, these practices of changing and washing are interlinked with the practice of cycling, but in this case, they seem to directly result from the practice of commuting (and getting sweaty) and, because of this, they have the potential to influence each other. Talk of these considerations were identified in the men’s data from all four of the cycling-related datasets (within this query about Work and Home specifically), and the men’s accounts of cycling were connected with other logistical or organisational concepts such as Use and Road, and themes of Transport, Take, Car and Minutes.

The practice of relaxing in the time and space on the commute was evident in data from both gender subgroups. This idea of taking time out, and using the commute as an opportunity to do so, is recognised as valuable to men and women in separating the practice of work with life at home, and in contributing to positive mental health.

‘ … *in terms of my emotional and mental health it’s, as I said, good thinking time, good processing time, calm down if I’ve had a stressful day at work, I find it’s really good wind down rather than sort of getting home still in a state … it’s a good sort of cooling down period’* (COBIM12).

*‘although you have to focus and concentrate while cycling I think you can relax and you can let the day just wash away and, so you don’t get home and you’re still thinking over and over about what happened at work,*’ (CCIF7).

This concept was predominantly identified within the cycling studies data, but it was also evident in other studies and there was talk about relaxing on the bus or train, driving the car and walking as well as cycling. In this way, these practices of relaxing could also be seen as ‘resulting practices’ – relaxing on the commute or as a positive outcome of a commute. Men and women similarly spoke about using the time and space to ‘wind down’, ‘switch off’ and spoke about ‘clearing’ their heads and ‘distancing’ themselves from work with reference to their own emotional and mental health. The other wellbeing-related concept spoken about by women and men was about energy, feeling energised or revitalised.

*‘Well it’s much nicer in the morning because you arrive at work kind of having done like a minor workout, you feel much more refreshed, much more energised’, (COCIM11).*

*‘ … when I cycled in I just felt really alive, full of energy despite the turmoils, and I really liked that, yeah it just makes you feel good’. (CCIF25).*

These examples of talk about feeling energised were largely made in the context of active travel (cycling and walking).

#### Discourses about practices: internal and external framing of travel experiences

A gendered difference apparent in the outputs from stage one was that some concepts were more dominant for either men or women. One difference in particular was the relevance of the concept Feel. In the women’s dataset, references to how one feels, or feelings about how women are perceived or identified were evident throughout the excerpts linked to the concept of Look, but overtly supported in the direct concept connection between Cycle and Feel. Women spoke explicitly about how modes of transport make them feel, predominantly during and after travel (for example health benefit and harms). Most of the excerpts refer to safety (mainly regarding risk of accidents, but in some cases other aspects of health) and confidence or competence as a cyclist.

*‘I like cycling but I don’t feel safe in a great many situations’ (COCIF15).*

‘*I don’t know, I like to, I go to cycle shops, I always look at cycling information, I would be, I just feel like a cyclist’ (CCIF27).*

This direct link between Cycle and Feel was not present for men and the few references to ‘feel’ indicated that the men spoke more about their feelings in a very general way, for example,

‘*Other times, I just wake up and I just, sometimes I just don’t feel like it, just either physically or mentally I just think oh, you know, I think I want to just sit on the bus today.’ (COCIM2).*

For men, Feel was not linked to Cycle (and was a less important concept overall (Additional file 2: Table B). Instead, Cycle clustered closer to Road. The associated excerpts indicate that the men did refer to concerns of safety, but in the context of the conditions being dangerous (for example a dangerous road, or dangerous weather) rather than directly referring to how they ‘feel’ about the situation.

‘ … *but the road is pretty dangerous you know, so that’s the reason I don’t cycle as much as I would like, in fact I don’t cycle at all’ (COAIM3)*

*‘Basically because enjoying cycling in the summer more so than in the winter with the weather and that … it can be quite dangerous when you’ve got ice and ruts and snow and that, yeah’ (COCIM6).*

These references to danger were largely made in the context of logistics, the routes they take to avoid the danger, and the differences in time taken for different routes.

‘*it became quite dangerous to use it because of the snow and ice and they weren’t gritting it, so I switched to the road and I even cycled in when we had the snow, because I could, and yeah, it just seems more direct, it’s just a much more direct route and the speed I go means that I’m not being harassed by cars too much*.’ (COAIM19).

This focus on organisation in the context of cycling is exemplified in the closely clustered themes with logistical theme labels assigned on the men’s map; Minutes, Road, Take.

The text excerpts that indicate this discursive difference were related to specific concept connections identified by the software (Feel and Cycle for women and Cycle and Road for men), and therefore could not be assumed to represent the dataset as a whole. In an effort to explore the presence of this phenomenon within the full dataset, we conducted a form of sensitivity analysis via Leximancer’s ‘user-defined concept’ function. This allowed us to explore the presence of two specific concepts of interest (Feel and Dangerous) across the full dataset.

The findings of this ‘sense-checking’ support our earlier finding. For example, in the context of other modes of travel and across the study datasets, women spoke more in a subjective way about how the situation made them feel, particularly in the context of safety; on the bus, driving with friends and walking, whereas the men’s data echoed the more objective framing evident in the cycling accounts:.

*‘Is awful. And I actually feel safer with a lot of my friends than I do my mum’. (YDGF4; Young Drivers, Focus group, Female, no.4 – talking about travelling in the car).*

*‘No, I wouldn’t like to walk about here. I just don’t feel safe enough’. (MGIF1).*

*‘Yeah, I would go on the bus but I wouldn’t go upstairs on a bus at night because I’ve had a few situations where there’s quite like dangerous people upstairs like …* ’ (OBIM15).

*‘ … and Allison Street, in particular, when you drive along at night, it’s not a safe place’*. (MGIM7).

Discourses about travel also differed by gender within the text excerpts connected to Look. Women’s highly connected accounts of Cyclists with Look were strongly expressed in the data. They referred to cyclist’s identity as they spoke about how cyclists look or the clothes they wear. Women referred to identity in the context of both negative and positive physical images,

‘*I think it’s possibly portrayed as a bit nerdy and people wearing, that’s my immediate, it’s people wearing nerdy outfits’ (CCFI23).*

*‘ … it is quite hard unless you’re quite fit to cycle up that hard hill … your heart rate would be high and you’d be getting quite hot, wouldn’t you and girls want to look pretty when they get to work, you know … it would be not the best thing to do before you get to work really … ’ (COCIF30).*

*‘And then I started seeing girls on their bikes in high heels and I think that’s a really lovely image...’ (CCIF17)*.

And for some women, cyclist’s identity was portrayed as one’s ability as a cyclist or definitions of the type of person a cyclist is, as exemplified in these accounts.

‘ … *people I know who cycle, yeah, I just kind of think they’re very healthy people* … ’ (CCIF5).

‘ *… people are trying to do it because they’re eco warriors or they’re only really fit and healthy’ (CCIF26).*

Evident across the pooled datasets, women referred to self-concepts such as how they look themselves or how they feel that they are perceived as a cyclist or as a user of shared travel environments such as the bus, or in the park:

‘ *… I’ve got front light, back light, a reflective jacket and I’ve even got a little flashing light on my helmet and … I heard someone saying, oh look, it’s a disco when I was cycling home, because everything was flashing. So I’m very, I’m definitely safety conscious ahead of any fashion consciousness, because I think I do look ridiculous when I’ve got all my winter gear on’(CCIF13)*.

‘*Whenever I’m on the bus and there’s someone with a buggy, I’ll help them on, and stuff, and I think that, that they have respect for you because they know, if you show it, then they know that you’re, you’re a respectful person*,’(OBGF21),

*‘ … I think also with the kids, round my area there’s a lot of kids who take over the park, and I don’t want to be the adult trying to learn how to ride a bike, you know what I mean? It’s all these factors, no I’ll go on a bus instead … ’(CCIF4).*

The talk about self-concepts and identity were rich in the excerpts of Look for women, and were much less evident in the men’s accounts of Look which, as the maps suggest, related more to ‘looking’ at the environment, the look of the place and area than relating to people.

## Discussion

### Gendered patterns - practices and discourses

The aim of this secondary data analysis was to identify gendered patterns in the context of travel practices. The text analytics outputs indicated that, on the surface, men and women’s transcripts seemed to contain largely similar topics of travel. This included home and work, despite only one of the primary studies investigating commuting, but also places such as school, and diverse travel modes, even though some studies focused on particular modes such as bus travel or cycling. However, our interpretative findings indicate gender differentiated experiences and travel narratives. These new understandings were not described in the original analyses of these datasets.

First, we found a gendered difference in how practices associated with commuting were ‘bundled together’ [19]. For women across our pooled studies, travel practice co-occurred with other practices that require travel such as combining commuting with the school run or getting grocery shopping done at specific times of the day. Social theories of practice highlight the importance of understanding in which way practices are rarely enacted in isolation but are enacted as part of other practices and with or for other people [17, 18]. The pooled women’s accounts provided plentiful empirical examples across settings and modes of multifunctional trips that allow them to navigate a complex world with synchronisation and coordination, but ultimately limit their choice of travel mode [13]. In contrast, these accounts were rare within the men’s data, who spoke more about commuting in a linear way (e.g. getting from work to home). This aligns with existing evidence that such gendered differences do occur and women are more likely to ‘trip-chain’ [11, 32], as a consequence of women in couples taking on the responsibility of the household [9, 11], particularly childcare [32, 33] and other unpaid work [34], or as a result of inequalities in the impact of life events on women [15]. Furthermore, women are more likely than men to travel with children or elderly relatives and carry shopping or a buggy [32], potentially necessitating more complex travel arrangements.

Second, in this pooled dataset, we identified a gendered difference in the way men and women talked about travel practice across contexts, particularly with reference to safety. Interestingly, despite many accounts found when exploring this further in the excerpts related to Feel, ‘safety’ was not identified as its own ‘concept’ or ‘theme’ in the automated text analytics. However, we found that women, more than men, spoke about themselves as practitioners; how they are identified, or identify themselves as an actor in travel; how they relate to socio-cultural norms of other actors of travel; and how the practice made them *feel* (for example as the ‘cyclist’, or the bus user). This talk about how women felt was particularly evident in accounts of danger, where their talk was internally framed, in terms of feelings of danger (things made them *feel* safe or unsafe), whereas men’s talk was externally framed, in terms of danger as a fact about the external world (things *are* dangerous).

Developing safe environments (for example, by segregating walkers and cyclists from traffic or reducing perceptions of crime) is indicated as an important consideration when developing environments to promote active travel [35]. However, our finding of a disproportionate concern between men and women over feelings of safety in the context of travel and mobility align with evidence that women report being more fearful than men in travel situations (twice as many women than men reported that they do not *feel* safe using public transport in London [36]). There are a number of reasons why these concerns over safety may be greater for women [32, 37, 38], but ultimately, these fears do impact on women’s travel decisions [37].

### Implication for practice and research

Two particular implications might arise from this analysis. Understanding that travel practices might be combined in varied ways rather than within linear journeys has been increasingly acknowledged in the context of transport policy in the UK (data that is not separated by gender) [4], but is still a relatively new way of addressing travel planning or infra-structural planning needs. Moreover, our study found that men and women combine travel practices in different ways – or even that some travel practices might in fact be linear whereas others co-occur as multifunctional trips (in our case, by gender). These important differences raise questions as to whether there are other situations, aside from travel, whereby practices sit together in different ways according to gender.

Differential narratives about concerns that shape travel, in our case safety and identity, also suggest that more nuanced approaches to encouraging active travel might be needed. For example, more nuanced framing as internal or person-centred versus external or environment-centred might address safety concerns in more meaningful ways for different population groups. While in our settings, or rather analysis, a dominant differential narrative appeared to be between men and women, this might also translate into differential concerns between ages or ethnic groups, or concerns about other important issues or mechanisms for active travel, such as accessibility or convenience [35]. Existing research into the effectiveness of message framing on health behaviours focuses largely on negative versus positive, or ‘loss’ versus ‘gain’ language, and suggests that further evidence is required to understand how we can get a consistent ‘framing effect’ to encourage healthy behaviour [39, 40]. To better understand the significance of these gendered discourses, it is important to evaluate campaigns and interventions that use gendered framing, and these investigations could provide important insights into how we can effectively communicate across genders to promote public health. Thinking about research and public engagement, these differences in how issues are articulated, could be considered when consulting the public about what they need. One implication is that survey questions asking about people’s ‘feelings’ of safety (rather than whether transport modes are safe) might under-estimate men’s concerns.

From a methodological standpoint, these insights have come from our response to calls to enhance the secondary use of qualitative data. We believe that this method has enhanced our understanding of gendered patterns in the context of travel. By broadening the evidence base, through combining datasets and reanalysing them together, we have become more aware of the challenges around quantifying qualitative data, but have ultimately gained insight into an important facet of gendered travel practice and discourses that were not identified in the original analyses.

### Strengths and limitations

As with any form of secondary data analysis, the reliability of the outputs is determined by the quality of the primary datasets. One way that we ensured this in our synthesis was by including datasets that we were familiar with, and could confirm the rigour of the original enquiry. Further, in an effort to conduct some form of sensitivity analysis, we coded the original excerpts to better understand the contexts from which they were derived. Given that these were transcripts of substantial length, they contained some level of context; this kind of reliability check might be more challenging with less rich data (such as social media posts). One particular limitation of the original datasets was that information about gender was limited to self-identification on a binary category of male/female. We could not include other identifications, or evidence about the gendered nature of interaction between interviewer/interviewees.

We also acknowledge that within the process of synthesising qualitative data, a fine balance exists between the strength and novelty of cross-context comparison and the limitation of losing contextual insights and richness of individual qualitative accounts. This process has highlighted key analytical similarities between machine learning and traditional qualitative techniques, in that both involve a systematic process of developing codes (or ‘concepts’ in Leximancer) and grouping them into higher order themes. However, some major differences in the direction of the analytical journey impart different limitations between the two approaches. We acknowledge that conventional techniques tend to work from the text up and provide contextual relevance from the beginning of the analysis and the option to discount frequently occurring linguistic terms that have little conceptual meaning. In contrast, semi-automated text analytics provides the higher order themes first. To make meaning of these themes, the researcher must return to the texts excerpts to understand what is meant by these ultimately linguistic labels. While these excerpts are conveniently identified as relevant by the software, they are, nonetheless, numerous. This requires substantial input from the researcher and may not be a speedy solution to qualitative data synthesis. Yet machine learning provides the benefit of showing all connections that exist in the data, many of which the researcher may not have the time (or the interest) to explore manually, and reduces the risk of missing important insights that may be initially less appealing to the researcher, and can make the synthesis of large qualitative datasets feasible.

## Conclusion

Current approaches to encouraging active living on a population level move away from individual behaviour interventions and experimental research designs, toward real-world natural experiments that can generate transferrable evidence. Such experiments increasingly include in-depth qualitative explorations of underlying mechanisms that bring about change [35, 41]. We acknowledge the abundance of transferrable evidence that lies dormant in these qualitative real-world accounts of active travel, along with evidence regarding other health-related behaviours, and questions have been raised as to how we make better use of these context specific findings to inform transferrable insights [42].

This study on social practices of travel contributes to existing evidence about travel as a gendered practice and found differential experiences and narratives of travel. Using data from context-specific qualitative studies, with the additional insight of comparing across contexts, we found similarities and some marked differences in the way that women’s travel practices bundle together as multi-functional journeys and men’s are joined in a more linear way. Furthermore, women’s and men’s talk of danger and safety highlight gender differential accounts of travel experience. These findings have important implications for those developing interventions to promote healthier travel practices. They suggest a need to consider how gender (and other social positions) might shape both practice articulations and discursive accounts of those practices.

## Supplementary information

**Additional file 1: Table A.** Six steps of Leximancer analysis.

**Additional file 2: Table B.** Top 25 most frequent and relevant concepts.

## Data Availability

The original transcripts from the primary research studies included in this secondary analysis were only accessible to the authors for the length and use of this project, and are therefore not available to third parties; the corresponding author can be of assistance to liaise with the original institutions which hold the data.
