# Supplementary material for: Gender and active travel: a qualitative data synthesis informed by machine learning
Source: Int J Behav Nutr Phys Act. 2019 Dec 21;16:135. doi: 10.1186/s12966-019-0904-4 (PMC6925863; doi:10.1186/s12966-019-0904-4)
Supplement: Supplementary file 1 — Additional file 1: Table A. Six steps of Leximancer analysis. [file 12966_2019_904_MOESM1_ESM.docx]

**Additional File 1**

| *Table A: Six steps of Leximancer analysis* |
| --- |
| 1. *Formatting transcripts*: Each anonymised transcript was formatted by two researchers (EH, CG) to ensure compatibility with the software. We had access to core, but limited, contextual information for each transcript and this information was used to develop a unique identification number (UIN) for each transcript. The UIN provided important descriptors which were mainly the study setting, location (in a wider sense), gender, varied age ranges (easiest divided into adolescents, working age adults and retired), and were utilised in the second stage of the analysis. 2. *Classification for analysis*: Each transcript was ordered into sub-folders according to the participant’s gender. 3. *Automatic text processing and concept seed generation*: We conducted two individual analyses for the women’s and the men’s data. We ran the analysis by dialogue to enable the software to tag the data by the speaker’s identification number. This facilitated the identification of gender of speaker within interviews and mixed focus group transcripts. 4. *Concept editing*: Only automatically defined concepts were used and no tags or concepts were defined by the user. Identified concepts with little conceptual meaning were removed, such as ‘probably’, ‘obviously’ and ‘yeah’. Plurals of concepts or those with similar meaning were merged (bus and buses; car and cars; cycle and cycling; drive and driving; place and places; use and used; walk and walking) and the thesaurus settings were set to programme default. 5. *Concept coding*: The text was coded with ‘all discovered concepts’ that were identified automatically. The decision was made to ‘kill’ the name-like concept ‘interviewer’, to supress the processing of questions asked by the interviewer. In the men’s analysis, we ‘killed’ female descriptors that were identified as name-like concepts, to supress the processing of dialogue tagged as female, and vice versa for the women’s analysis. 6. *Output*: The social network (Gaussian) map was chosen over the topic network (linear) map to emphasise the conceptual context in which the words appear and maximise the discovery of indirect relationships. |
| *Source: Haynes et al 2019.* |
