# Supplementary material for: Gender and active travel: a qualitative data synthesis informed by machine learning
Source: Int J Behav Nutr Phys Act. 2019 Dec 21;16:135. doi: 10.1186/s12966-019-0904-4 (PMC6925863; doi:10.1186/s12966-019-0904-4)
Supplement: Supplementary file 2 — Additional file 2: Table B. Top 25 most frequent and relevant concepts. [file 12966_2019_904_MOESM2_ESM.docx]

**Additional File 2**

Table B: Top 25 most frequent and relevant concepts

| Women | | | Men | | |
| --- | --- | --- | --- | --- | --- |
| **Concept** | **Count** | **Relevance** | **Concept** | **Count** | **Relevance** |
| bus | 3178 | 100% | bus | 1722 | 100% |
| people | 1966 | 62% | people | 1007 | 58% |
| cycle | 1890 | 59% | cycle | 939 | 55% |
| use | 1698 | 53% | use | 928 | 54% |
| car | 1541 | 48% | car | 886 | 51% |
| time | 1523 | 48% | time | 823 | 48% |
| work | 1496 | 47% | walk | 770 | 45% |
| walk | 1458 | 46% | down | 633 | 37% |
| take | 1083 | 34% | work | 618 | 36% |
| drive | 1016 | 32% | bike | 569 | 33% |
| down | 992 | 31% | take | 552 | 32% |
| bike | 987 | 31% | drive | 546 | 32% |
| day | 915 | 29% | road | 458 | 27% |
| road | 893 | 28% | day | 458 | 27% |
| things | 875 | 28% | minutes | 432 | 25% |
| home | 826 | 26% | things | 430 | 25% |
| hour | 793 | 25% | home | 376 | 22% |
| school | 786 | 25% | doing | 366 | 21% |
| minutes | 722 | 23% | train | 365 | 21% |
| doing | 690 | 22% | place | 362 | 21% |
| nice | 664 | 21% | school | 362 | 21% |
| traffic | 631 | 20% | need | 322 | 19% |
| feel | 625 | 20% | coming | 321 | 19% |
| *to other concepts identified in the dataset (Smith 2006) | | | | | |
